# Phylogenomic resolution of marine to freshwater dinoflagellate transitions

**DOI:** 10.1093/ismejo/wraf031

**Published:** 2025-02-21

**Authors:** Mahara Mtawali, Elizabeth C Cooney, Jayd Adams, Joshua Jin, Corey C Holt, Patrick J Keeling

**Affiliations:** Department of Botany, University of British Columbia, Vancouver V6T 1Z4, British Columbia, Canada; Department of Botany, University of British Columbia, Vancouver V6T 1Z4, British Columbia, Canada; Department of Botany, University of British Columbia, Vancouver V6T 1Z4, British Columbia, Canada; Department of Botany, University of British Columbia, Vancouver V6T 1Z4, British Columbia, Canada; Department of Botany, University of British Columbia, Vancouver V6T 1Z4, British Columbia, Canada; Hakai Institute, Heriot Bay, British Columbia, Canada; Department of Botany, University of British Columbia, Vancouver V6T 1Z4, British Columbia, Canada

**Keywords:** dinoflagellates, phylogenomics, marine-freshwater transition, single-cell transcriptomics

## Abstract

Dinoflagellates are an abundant and diverse group of protists that inhabit aquatic environments worldwide. They are characterized by numerous unique cellular and molecular traits, and have adapted to an unusually broad range of life strategies, including phototrophy, heterotrophy, parasitism, and all combinations of these. For most microbial groups, transitions from marine to freshwater environments are relatively rare, as changes in salinity are thought to lead to significant osmotic challenges that are difficult for the cell to overcome. Recent work has shown that dinoflagellates have overcome these challenges relatively often in evolutionary time, but because this is mostly based on single gene trees with low overall support, many of the relationships between freshwater and marine groups remain unresolved. Normally, phylogenomics could clarify such conclusions, but despite the recent surge in data, virtually no freshwater dinoflagellates have been characterized at the genome-wide level. Here, we generated 30 transcriptomes from cultures and single cells collected from freshwater environments to infer a robustly supported phylogenomic tree from 217 conserved genes, resolving at least seven transitions to freshwater in dinoflagellates. Mapping the distribution of ASVs from freshwater environmental samples onto this tree confirms these groups and identifies additional lineages where freshwater dinoflagellates likely remain unsampled. We also sampled two species of *Durinskia*, a genus of “dinotoms” with both marine and freshwater lineages containing *Nitzschia*-derived tertiary plastids. Ribosomal RNA phylogenies show that the host cells are closely related, but their endosymbionts are likely descended from two distantly-related freshwater *Nitzschia* species that were acquired in parallel and relatively recently.

## Introduction

The transition between marine and freshwater environments was once assumed to be relatively unimportant in microbial species due to their large population sizes [1], high genetic variability [2], and potential for rapid dispersal [3, 4]. Morphological identification seemed to support this, with many taxa identified as the same species documented in both environments. However, molecular identification has since revealed this is not the case, as many morphologically identical specimens were found to be distinct species, and freshwater clades were found to be phylogenetically discrete from marine clades across diverse lineages. This transition is now recognized as one of the less frequent ecological shifts in the microbial world, and recent transitions are particularly rare [5]. Although this generally holds true across diverse microbes, there are exceptions, and dinoflagellates are one group where the transition appears more common [6, 7]. Dinoflagellates are abundant in most aquatic environments, and morphologically and trophically diverse [8]. Their diversification was likely driven by multiple environmental factors, and osmoregulation is argued to be among them [2]. Dinoflagellates are therefore not only ecologically significant in general, but also relevant models for evolutionary and ecological transitions.

Our current understanding of marine to freshwater transitions in dinoflagellates is based entirely on ribosomal RNA (rRNA) gene phylogenies [6, 9–13], where ancient relationships are mostly unsupported. The most comprehensive studies on the topic include a high degree of diversity, but have weak deep-branch node support - many freshwater groups appear to fall into discrete clades, but the rRNA data lack the resolving power to exclude alternatives where there are fewer transitions to freshwater [6, 9]. Phylogenomic analysis can often remedy this issue, and over 100 recently availability dinoflagellate transcriptomes have been used to resolve other evolutionary transitions [14]. But, incredibly, almost all available dinoflagellate genomic or transcriptomic data come from marine species, with the exception of two transcriptomes from *Apocalathium aciculiferum* collected from fresh and slightly brackish waters [15], sequenced as part of the Marine Microbial Eukaryote Transcriptome Sequencing Project [16].

Here, we have sequenced transcriptomes from 30 different freshwater dinoflagellate single cells and cultures, including representatives from many of the clades previously identified in rRNA gene phylogenies [6, 9], to test the phylogenetic distribution of freshwater dinoflagellates among marine clades. Cultures from several genera were obtained from the Canadian Centre for the Culture of Microorganisms and their cDNA was sequenced from TRIzol™ RNA extractions. Single cells were also isolated from freshwater environments, and RNA was extracted for single cell sequencing (Table S1 and Fig. S1) [17]. Cells were identified via phylogenetic affinity to dinoflagellate 18S rRNA gene sequences recovered from Genbank (Fig. S2). A dinoflagellate phylogeny was generated from an alignment of 217 conserved genes containing the 21 highest-coverage representatives across all collected groups, each represented by a subset of the genes used to generate the tree (Table S1). Further investigation was performed on diatom-bearing *Durinskia* samples, using 18S rRNA gene phylogenies to determine the relationship and environmental origins of their diatom endosymbionts. For methodologies, see Supplementary Methods.

**Figure 1:**
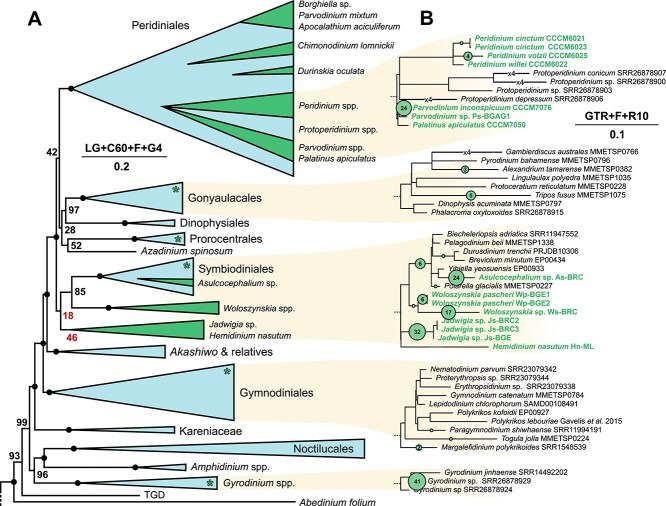
Distribution of freshwater transitions across dinoflagellates. A) Schematic of the maximum likelihood phylogeny of 80 dinoflagellates generated from an alignment of 50 503 positions from 217 conserved genes, and 1000 ultrafast bootstraps. Green and blue represent freshwater and saltwater clades, respectively, with green asterisks on clades either known to contain freshwater species that were not sampled in this study, or where currently unsampled freshwater diversity is indicated in panel B. Numbers at nodes are bootstrap support values, with black dots representing 100. Red, underlined values highlight weakly-supported nodes that may represent anywhere between one and three separate freshwater transitions. The model used to generate the phylogeny is shown with a scale bar for estimated amino acid substitutions per site. TGD (Tsuruoka green dinoflagellate) is an unnamed lineage [23]. The complete tree with all 87 taxa is shown in Fig. S3. B) Distribution of ASVs recovered from 10 freshwater datasets mapped onto an 18S rRNA gene reference dataset (Fig. S4) where the tree topology was constrained to the phylogenomic tree in Fig. S3. The clades with the highest frequency of mapped ASVs are shown, as are clades of predominantly marine taxa where ASVs suggest freshwater taxa should be found (the complete reference tree with all mapped ASVs is shown in Fig. S4). Freshwater taxa are shown in green; samples from the present study are bolded. Green circles represent the number of ASVs mapped onto the corresponding branch; numbers are shown for select branches. Shortened branches are labeled with the factor by which they were reduced. The model used to generate the tree is shown with a scale bar of estimated nucleotide substitutions per site.

The resulting tree reveals at least seven independent marine to freshwater transitions (Fig. 1A and Fig. S2). Despite our transcriptome sampling being taxonomically limited compared to rRNA gene sampling of freshwater species, the lineages overlap [9, 10], and the support for the phylogenomic tree is very high, making the two approaches complimentary. Most freshwater clades are fully-supported (or very highly-supported in the case of *Peridinium*), with the exception of a few genera branching close to the Symbiodiniales: the union of *Jadwigia* and *Hemidinium* and their overall position in the tree lack support, leaving these relationships unresolved (Fig. 1A and Fig. S3). However, we interpret this to suggest that a *Jadwigia* + *Hemidinium* transition may be independent from a *Woloszynskia* transition, and depict it here as such (Fig. 1A). Within the Peridiniales clade, the marine *Protoperidinium* is nested within freshwater *Peridinium,* suggesting a possible transition to saltwater from freshwater (Fig. S3), but it is also possible that further taxon sampling will reveal this to be an ancestrally marine group with multiple transitions to freshwater. We generally interpret the ancestral state to be marine, but it should be noted that polarizing these transitions is difficult without very thorough sampling.

We tested the environmental distribution of the freshwater clades found here and examined the possibility that more freshwater transitions remain unsampled by mapping the phylogenetic distribution of dinoflagellate-related rRNA genes from freshwater 18S rRNA gene amplicon sequencing surveys (Fig. 1B and Fig. S4). Most sequences isolated from freshwater datasets branched with freshwater clades already represented in our reference tree, but a few clustered within marine clades, some of which will represent freshwater lineages identified in previous analyses that are absent from our phylogenomic data [6, 9–13], whereas others likely represent freshwater clades that remain unidentified.

One lineage of particular interest is *Durinskia*, a member of the “dinotom” dinoflagellates, which contain diatom-derived tertiary plastids [18]. Whereas the dinotoms are all closely related, their endosymbionts descend from several distinct diatom subgroups, originating multiple times, and this has been observed in both fresh and saltwater species [19, 20]. The genus *Durinskia* contains both fresh and saltwater species with diverse *Nitzschia*-derived plastids [19]. *Nitzschia* is itself a lineage where multiple marine-freshwater transitions have taken place, as it has in at least one other diatom lineage [21], raising the intriguing possibility that the dinotoms replaced their plastids after transitioning to freshwater, perhaps multiple times.

**Figure 2:**
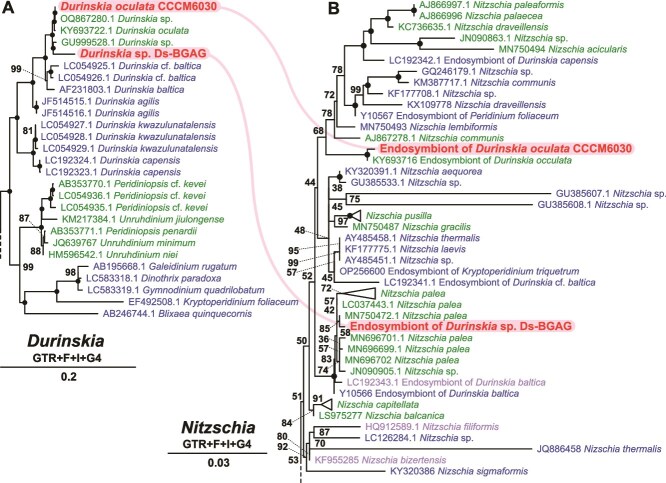
Phylogenetic relationships between hosts and endosymbionts for two species of *Durinskia* sampled in this study. Trees are (A) a dinoflagellate 18S rRNA gene tree and (B) a subset of a *Nitzschia* 18S rRNA gene tree, showing the host and endosymbiont positions, respectively (the complete *Nitzschia* tree is shown in Figs. S5). Freshwater, saltwater, and brackish lineages are shown in green, blue, and purple, respectively. Sequences reported in the present study are coloured red, highlighted, and bolded. Numbers at nodes show bootstrap values with a black dot representing 100. The model used to generate the phylogeny is shown with a scale bar for the estimated number of nucleotide substitutions per site.

We sampled two freshwater *Durinskia*; *D. oculata* from culture, and a *Durinskia* sp. single cell that did not yield enough data for phylogenomics but did yield 18S rRNA gene sequences from both host and endosymbiont. To test whether these species share a common freshwater ancestor that transitioned long ago or recently, and whether they retain ancestral marine plastids or new freshwater plastids, we analyzed the phylogenetic position of both host and endosymbiont using 18S rRNA genes. Both hosts group closely with existing 18S rRNA gene sequences from *D. oculata* and other freshwater strains (Fig. 2A). By contrast, the endosymbionts are distantly related: the endosymbiont of *D. oculata* CCCM6030 branches closely with that of *D. oculata* from the Vltava River (KY693716), and not near the endosymbiont of their closest marine relative, *D. baltica,* and the endosymbiont of *Durinskia* sp. Ds-BGAG falls within the free-living *Nitzschia palea* clade (Fig. 2B and Fig. S5). Overall, it appears that the dinotom host transitioned once from marine to freshwater, but its descendants replaced their endosymbionts with two different freshwater diatoms. The question of whether the presence of osmotic diversity in *Nitzschia* facilitated the freshwater transition of *Durinskia* merits further exploration.

Overall, these data confirm that dinoflagellates are adept at making the difficult transition from marine to freshwater environments. Like many planktonic protists, dinoflagellates have long inhabited estuarine and sea ice habitats, and have thus evolved adaptations for fluctuating salinities. However, dinoflagellates may exhibit a particularly rapid DNA and RNA synthesis response, enabling them to maintain metabolic requirements more effectively [22]. This adaptation may help explain the frequency with which this group has invaded freshwater environments. We also identified evolutionary implications for recent plastid origins, and evidence that further marine to freshwater transitions remain to be discovered, altogether consistent with the idea that dinoflagellates are an important model for this rare ecological transition.

## Supplementary Material

Supplementary_wraf031

## Data Availability

The data from this article are available in the Supplementary Information, on Genbank (PQ793387-PQ793418), and in the SRA database (PRJNA1183848). Nucleotide and translated amino acid assemblies are at https://doi.org/10.5683/SP3/JCIEYV.

## References

[1] Giovannoni SJ, Stingl U. Molecular diversity and ecology of microbial plankton. *Nature* 2005;437:343–8. 10.1038/nature04158 16163344

[2] Rengefors K, Annenkova N, Wallenius J et al. Population genomic analyses reveal that salinity and geographic isolation drive diversification in a free-living protist. *Sci Rep* 2024;14:1–13. 10.1038/s41598-024-55362-5 38424140 PMC10904836

[3] Falkowski PG, Fenchel T, Delong EF. The microbial engines that drive earth’s biogeochemical cycles. *Science* 2008;320:1034–9. 10.1126/science.1153213 18497287

[4] Xu X, Wang N, Lipson D et al. Microbial macroecology: In search of mechanisms governing microbial biogeographic patterns. *Glob Ecol Biogeogr* 2020;29:1870–86. 10.1111/geb.13162

[5] Logares R, Bråte J, Bertilsson S et al. Infrequent marine-freshwater transitions in the microbial world. *Trends Microbiol* 2009;17:414–22. 10.1016/j.tim.2009.05.010 19726194

[6] Žerdoner Čalasan A, Kretschmann J, Gottschling M. They are young, and they are many: dating freshwater lineages in unicellular dinophytes. *Environ Microbiol* 2019;21:4125–35. 10.1111/1462-2920.14766 31369197

[7] Jamy M, Biwer C, Vaulot D et al. Global patterns and rates of habitat transitions across the eukaryotic tree of life. *Nat Ecol Evol* 2022;6:1458–70. 10.1038/s41559-022-01838-4 35927316 PMC9525238

[8] Schnepf E, Elbrächter M. Nutritional strategies in dinoflagellates: a review with emphasis on cell biological aspects. *Eur J Protistol* 1992;28:3–24. 10.1016/S0932-4739(11)80315-9 23194978

[9] Logares R, Shalchian-Tabrizi K, Boltovskoy A et al. Extensive dinoflagellate phylogenies indicate infrequent marine-freshwater transitions. *Mol Phylogenet Evol* 2007;45:887–903. 10.1016/j.ympev.2007.08.005 17928239

[10] Takahashi K, Moestrup Ø, Jordan RW et al. Two new freshwater woloszynskioids *Asulcocephalium miricentonis* gen. Et sp. nov. and *Leiocephalium pseudosanguineum* gen. Et sp. nov. (Suessiaceae, Dinophyceae) lacking an apical furrow apparatus. *Protist* 2015;166:638–58. 10.1016/j.protis.2015.10.003 26599726

[11] Annenkova NV, Hansen G, Rengefors K. Closely related dinoflagellate species in vastly different habitats–an example of a marine–freshwater transition. *Eur J Phycol* 2020;55:478–89. 10.1080/09670262.2020.1750057

[12] Takano Y, Yamaguchi H, Inouye I et al. Phylogeny of five species of Nusuttodinium gen. Nov. (Dinophyceae), a genus of unarmoured Kleptoplastidic dinoflagellates. *Protist* 2014;165:759–78. 10.1016/j.protis.2014.09.001 25460229

[13] Onuma R, Watanabe K, Horiguchi T. *Pellucidodinium psammophilum* gen. & sp. nov. and *Nusuttodinium desymbiontum* sp. nov. (Dinophyceae), two novel heterotrophs closely related to kleptochloroplastidic dinoflagellates. *Phycologia* 2015;54:192–209. 10.2216/14-103.1

[14] Cooney EC, Holt CC, Hehenberger E et al. Investigation of heterotrophs reveals new insights in dinoflagellate evolution. *Mol Phylogenet Evol* 2024;196:108086–14. 10.1016/j.ympev.2024.108086 38677354

[15] Logares R, Boltovskoy A, Bensch S et al. Genetic diversity patterns in five protist species occurring in lakes. *Protist* 2009;160:301–17. 10.1016/j.protis.2008.10.004 19162540

[16] Keeling PJ, Burki F, Wilcox HM et al. The marine microbial eukaryote transcriptome sequencing project (MMETSP): illuminating the functional diversity of eukaryotic life in the oceans through transcriptome sequencing. *PLoS Biol* 2014;12:1–6. 10.1371/journal.pbio.1001889 PMC4068987 24959919

[17] Picelli S, Faridani OR, Björklund ÅK et al. Full-length RNA-seq from single cells using smart-seq2. *Nat Protoc* 2014;9:171–81. 10.1038/nprot.2014.006 24385147

[18] Hehenberger E, Imanian B, Burki F et al. Evidence for the retention of two evolutionary distinct plastids in dinoflagellates with diatom endosymbionts. *Genome Biol Evol* 2014;6:2321–34. 10.1093/gbe/evu182 25172904 PMC4217693

[19] Yamada N, Sym SD, Horiguchi T. Identification of highly divergent diatom-derived chloroplasts in dinoflagellates, including a description of *Durinskia kwazulunatalensis* sp. nov. (Peridiniales, Dinophyceae). *Mol Biol Evol* 2017;34:1335–51. 10.1093/molbev/msx054 28333196

[20] Horiguchi T, Takano Y. Serial replacement of a diatom endosymbiont in the marine dinoflagellate *Peridinium quinquecorne* (Peridiniales, Dinophyceae). *Phycol Res* 2006;54:193–200. 10.1111/j.1440-1835.2006.00426.x

[21] Roberts WR, Ruck EC, Downey KM et al. Resolving marine-freshwater transitions by diatoms through a fog of gene tree discordance. *Syst Biol* 2023;72:984–97. 10.1093/sysbio/syad038 37335140

[22] Skarlato S, Filatova N, Knyazev N et al. Salinity stress response of the invasive dinoflagellate *Prorocentrum minimum*. *Estuar Coast Shelf Sci* 2018;211:199–207. 10.1016/j.ecss.2017.07.007

[23] Sarai C, Tanifuji G, Nakayama T et al. Dinoflagellates with relic endosymbiont nuclei as models for elucidating organellogenesis. *Proc Natl Acad Sci USA* 2020;117:5364–75. 10.1073/pnas.1911884117 32094181 PMC7071878

