# Supplementary material for: Phylogenomic resolution of marine to freshwater dinoflagellate transitions
Source: ISME J. 2025 Feb 21;19(1):wraf031. doi: 10.1093/ismejo/wraf031 (PMC11937819; doi:10.1093/ismejo/wraf031)
Supplement: Supplementary_wraf031 [file supplementary_wraf031.pdf]

Supplementary Methods

Culture extractions and single cell isolation and sequencing

Freshwater dinoflagellate cultures were obtained from the Canadian Centre for the Culture of Microorganisms (CCCM) at UBC (**Table S1**). To extract mRNA from each culture, TRIzol™ LS was used, following the approach of Cho et al. [1] with an alteration: 500uL of 100% isopropanol was used to precipitate the RNA pellet after Chloroform layer separation, and 20 uL of ultrapure water was used for final resuspension. Multiple extractions were performed for each culture, sometimes altering light or temperature conditions slightly (by placing cultures in a dim 16°C incubator, a lit 16°C incubator, or left at room temperature near a window) to maximize transcriptome coverage.

Dinoflagellate single cells were collected from various freshwater habitats in western British Columbia, Canada (see **Table S1** for exact locations). Water samples were examined under a Leica DMIL inverted microscope and cells of interest were isolated using a stretched microcapillary pipette. Cells were washed 2-3 times in filtered (0.2µm) water from the same sample, videoed using a Sony A7r III (**Figure S1**), and placed into lysis buffer (Triton-X, RNaseOUT) [2]. These samples were then frozen at -70°C until cDNA generation could take place. To supplement TRIzol™ LS extractions, single cells were collected from some cultures using this same method.

Sequencing and transcriptome assembly

The SmartSeq2 protocol was used to generate cDNA from all samples [2, 3]. cDNA libraries were generated by the Sequencing and Bioinformatics Consortium at University of British Columbia using Illumina DNA Prep and sequenced on the Nextseq platform. Resulting forward and reverse raw reads were trimmed with Cutadapt v3.2 [4] and assembled using rnaSPAdes [5]. TransDecoder v5.5.0 was used to identify open reading frames and translate nucleotide sequences to amino acids [6], and BLASTp [7] was used to search sequences against the UniProt/Swiss-prot database [8] using an e-value threshold of 1e-5 so that sequence extensions could be identified and trimmed away. In cases where multiple transcriptomes were generated from the same culture, transcriptomes were concatenated and subsequently treated as a single transcriptome for all downstream analyses. All concatenated transcriptome assemblies can be found at https://doi.org/10.5683/SP3/JCIEYV, and raw reads are available in the SRA database under the project ID PRJNA1183848.

Phylogenomic and rRNA gene analyses

Searches were performed on peptide transcriptomes for orthologs of 263 conserved genes [9] using BLASTp, with an e-value threshold of 1e-20, only keeping sequences with more than 50% query coverage. Resulting hits were combined with existing alignments of their respective genes using MAFFT v7.481 [10]. Alignments were trimmed using trimAl v1.2 (-gt 0.8) and sequences were either verified or identified as contaminants or paralogs for removal via visual inspection of single-gene trees generated with FastTree v 2.1.11 [11]. One representative sequence was selected per transcriptome for each gene and SCaFoS v4.55 was used to select a subset of OTUs for the final tree, omitting genes present in ≤60% of these OTUs. Several of the lowest-coverage transcriptomes from freshwater clades were omitted to improve the stability of the analysis overall, leaving 21 new freshwater transcriptomes in the analysis. The final concatenated alignment (available at https://doi.org/10.5683/SP3/JCIEYV) consisting of 87 total OTUs, 217 conserved genes, and 50,503 sites was used to generate a maximum likelihood (ML) phylogeny in IQ-TREE v1.6.12 [12], using the empirical profile mixture model LG+C60+F+G4 [13] and 1000 ultrafast bootstraps [14].

Sequences of 18S rRNA genes were extracted from all transcriptomes using Barrnap v0.9 (https://github.com/tseem ann/barrnap) and the most complete sequence was selected to represent each transcriptome. Sequences were searched against Genbank using BLASTn to recover highly similar sequences. Hits with the highest identity to our sequences were aligned along with our sequences to a curated collection of diverse dinoflagellate 18S rRNA gene sequences using MAFFT. This alignment was trimmed using trimal (-gt 0.3), and used to generate a ML tree in IQ-TREE with the model GTR+F+I+G4 and 1000 ultrafast bootstraps.

We used the phylogenetic affinity portrayed in this tree to identify our cells collected from the environment to the genus or species level. We refer to all cultured samples by the taxonomic names they were given at CCCM, except in cases where these names were documented in Algaebase [15] as synonyms of a different, currently accepted name. For these cases, we refer to the sample using the currently accepted synonym (**Table S1**).

For *Durinski* samples, sequences from the endosymbiont were also identified. ML phylogenies were generated for both dinoflagellates and *Nitzschia* by curating collections of relevant genes from NCBI. For *Nitzschia*, only samples with available collection site information were used, so that cells could be identified as freshwater, saltwater, or brackish water species. Sequences were aligned with MAFFT, and trimmed with trimAl (-gt 0.3). Both trees were generated in IQ-TREE using the model GTR+F+I+G4 and visualized using FigTree v1.4.4 (https://github.com/rambaut/figtree). All 18S rRNA gene sequences are available from Genbank under accession PQ793387-PQ793418.

Evolutionary Placement Algorithm

To assess whether other freshwater dinoflagellate sequences showed similar phylogenetic distributions, and to detect freshwater groups not sampled in this study, 442 Amplicon Sequence Variants (ASVs) from 10 freshwater metabarcoding datasets were downloaded from metaPR2 [16] and placed onto a reference tree using an evolutionary placement algorithm along with 165 long-read metabarcodes from 5 additional datasets [17]. Full-length dinoflagellate 18S rRNA gene sequences were first aligned with MAFFT, before a ML tree, constrained to the corresponding phylogenomic topology, was generated using IQ-TREE and the recommended substitution model, GTR+F+R10. Freshwater dinoflagellate ASVs (herein used to describe both short and long reads) were added to the alignment with MAFFT (--addfragment, --keeplength; or --add for near full-length sequences) resulting in a total of 694 sequences and 1955 positions. The aligned ASVs were then separated from the reference sequences to produce two distinct alignments. Finally, EPA-NG v.0.3.8 was used to place the query sequences on the reference tree following dynamic heuristic preplacement and the default accumulated likelihood weight threshold [18]. A minimum mass threshold value of 0.7 was used to remove spurious placements using Gappa [19] resulting in 314 high confidence placements. A maximum of seven placements per sequence were displayed on the tree (i.e., the EPA-NG default) using iTOL (https://itol.embl.de).

Table S1. Collection information and identification for all isolated single cells and culture-derived freshwater dinoflagellates. Bar graphs show the presence of genes from the 263 gene set for all species and from the 217 gene subset used to generate the ML phylogeny in samples that were included in this analysis. Blue bars represent ortholog presence while pink represents missing orthologs. Taxa in red are taxonomically reassigned from their original names based on classification updates; these taxa were originally referred to at CCCM as *Peridinium elpatiewsky* (6034), *Peridinium inconspicuum* (7076), and *Peridinium eximium* (6023).

Source	Taxon	ID	Isolation Date	Collection Site
Environmental	*Asulcocephalium* sp.	As-BRC	15-Mar-22	UBC Biodiversity Research Centre courtyard trough
	Chimonodinium lomnickii	Cl-JP	28-Jan-22	Jericho Pond, Vancouver, BC
	Durinskia sp.	Ds-BGAG	26-Oct-22	UBC Botanical Garden Asian Garden pond
	Hemidinium nasutum	Hn-ML	21-Sep-21	Mud Lake, Quadra Island
	Jadwigia sp.	Js-BGE	03-Feb-22	UBC Botanical Garden entrance pond
	Jadwigia sp.	Js-BRC1	08-Mar-23	UBC Biodiversity Research Centre courtyard trough
	Jadwigia sp.	Js-BRC2	08-Mar-23	UBC Biodiversity Research Centre courtyard trough
	Jadwigia sp.	Js-BRC3	08-Mar-23	UBC Biodiversity Research Centre courtyard trough
	Parvodinium sp.	Ps-BGAG1	26-Oct-22	UBC Botanical Garden Asian Garden pond
	Peridinium sp.	Ps-JP	28-Jan-22	Jericho Pond, Vancouver, BC
	Peridinium sp.	Ps-BGAG2	26-Oct-22	UBC Botanical Garden Alpine Garden pond
	Peridinium sp.	Ps-BGAG3	26-Oct-22	UBC Botanical Garden Alpine Garden pond
	Woloszynskia pascheri	Wp-BRC	18-Jan-22	UBC Biodiversity Research Centre courtyard trough
	Woloszynskia pascheri	Wp-BGE1	03-Feb-22	UBC Botanical Garden entrance pond
	Woloszynskia pascheri	Wp-BGE2	03-Feb-22	UBC Botanical Garden entrance pond
	Woloszynskia sp.	Ws-JP1	28-Jan-22	Jericho Pond, Vancouver, BC
	Woloszynskia sp.	Ws-JP2	28-Jan-22	Jericho Pond, Vancouver, BC
	Woloszynskia sp.	Ws-BRC	15-Mar-22	UBC Biodiversity Research Centre courtyard trough
Cultures	*Apocalathium* sp.	CCCM6010	--	Canadian Centre for the Culture of Microorganisms
	Borghiella sp.	CCCM6014	--	Canadian Centre for the Culture of Microorganisms
	Chimonodinium lomnickii	CCCM6027	--	Canadian Centre for the Culture of Microorganisms
	Durinskia oculata	CCCM6030	--	Canadian Centre for the Culture of Microorganisms
	Palatinus apiculatus	CCCM7050	--	Canadian Centre for the Culture of Microorganisms
	Parvodinium elpatiewsky	CCCM6034	--	Canadian Centre for the Culture of Microorganisms
	Parvodinium inconspicuum	CCCM7076	--	Canadian Centre for the Culture of Microorganisms
	Parvodinium mixtum	CCCM6017	--	Canadian Centre for the Culture of Microorganisms
	Peridinium cinctum	CCCM6021	--	Canadian Centre for the Culture of Microorganisms
	Peridinium cinctum	CCCM6023	--	Canadian Centre for the Culture of Microorganisms
	Peridinium volzii	CCCM6025	--	Canadian Centre for the Culture of Microorganisms
	Peridinium willei	CCCM6022	--	Canadian Centre for the Culture of Microorganisms
Durinskia symbionts	*Nitzschia palea*	Ds-BGAGsym	26-Oct-22	UBC Botanical Garden Asian Garden pond
	Nitzschia sp.	CCCM6030sym	--	Canadian Centre for the Culture of Microorganisms

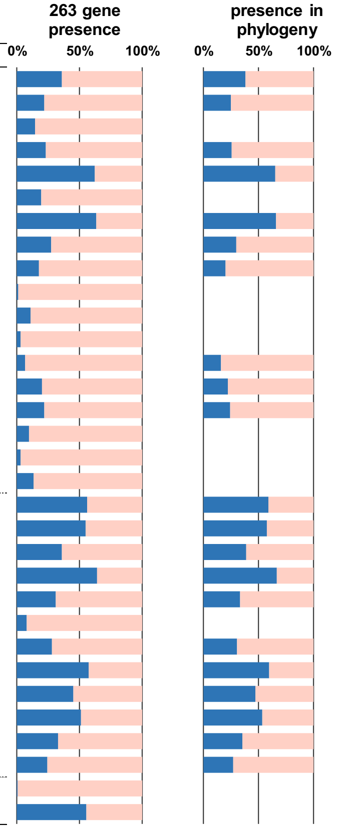

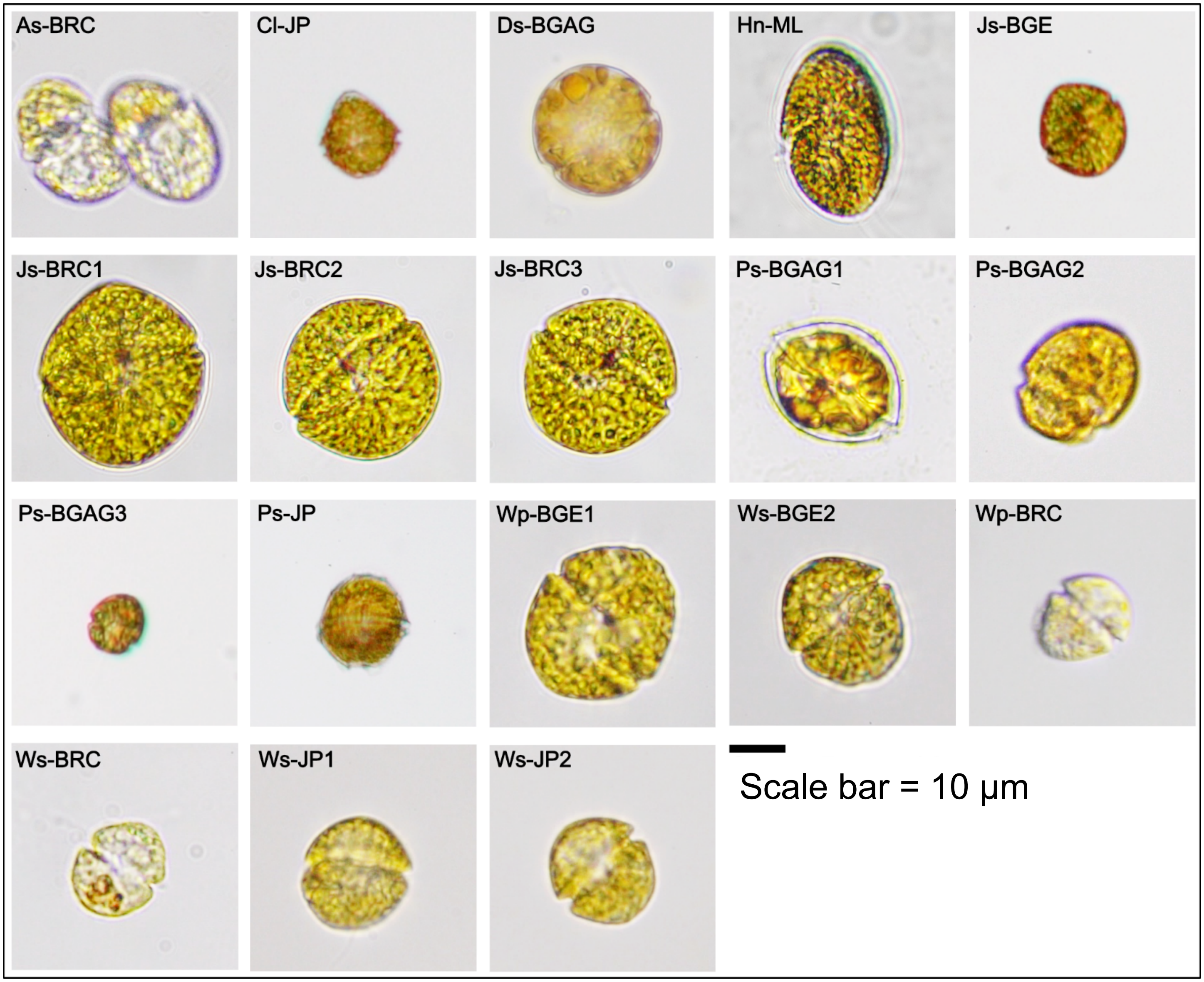

Figure S1. Light micrographs of single cells isolated from freshwater habitats. See **Table S1** for taxonomic information.

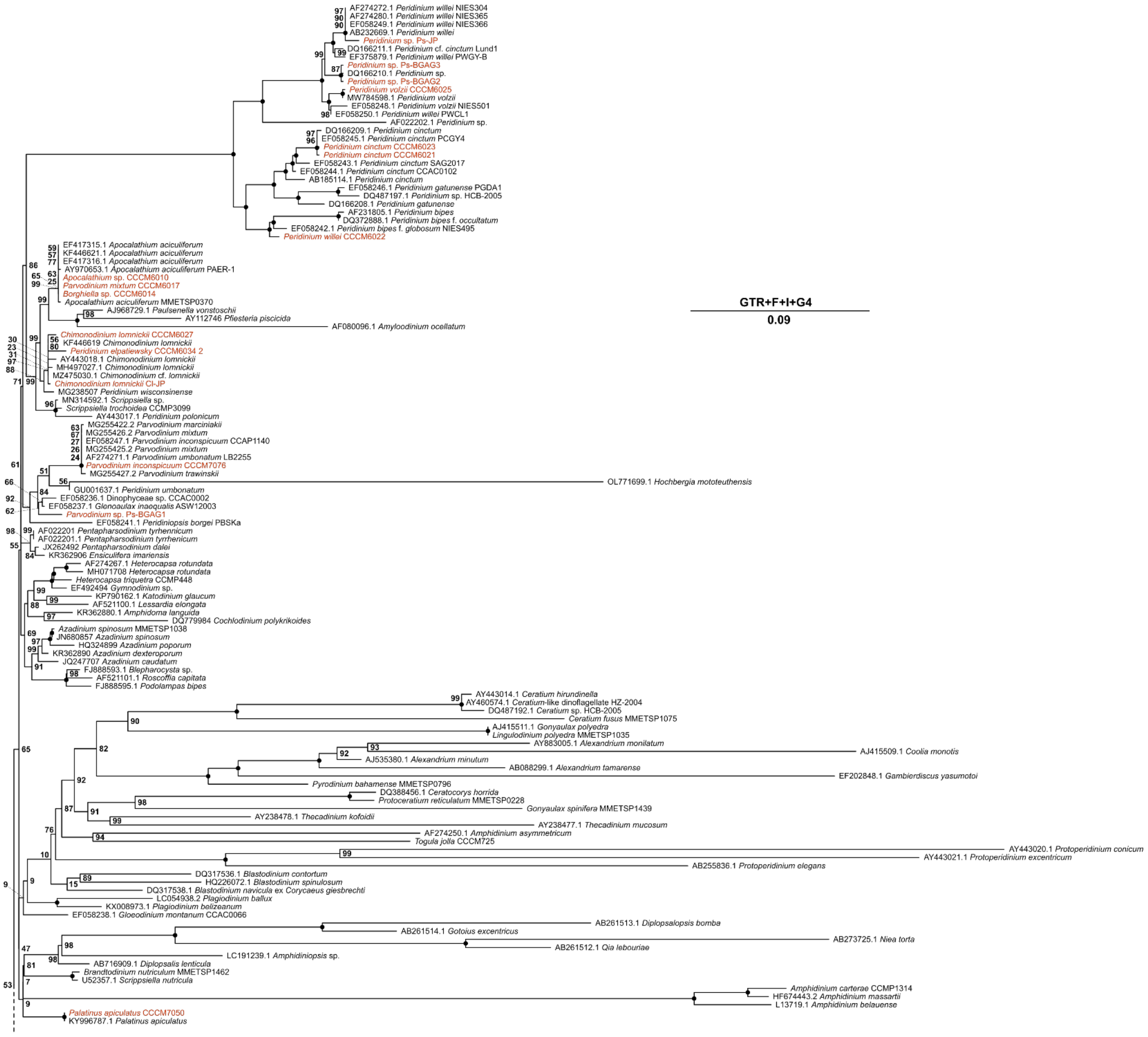

Figure S2. Maximum likelihood 18S rRNA gene phylogeny of dinoflagellates with sequences from the present study highlighted in orange. Node numbers represent bootstrap values, with 100 signified by a black dot. The model used to generate the tree is shown above a scale bar showing the estimated number of amino acid substitutions per site. Broken branches are depicted at half their original length. Figure is continued on the next page.

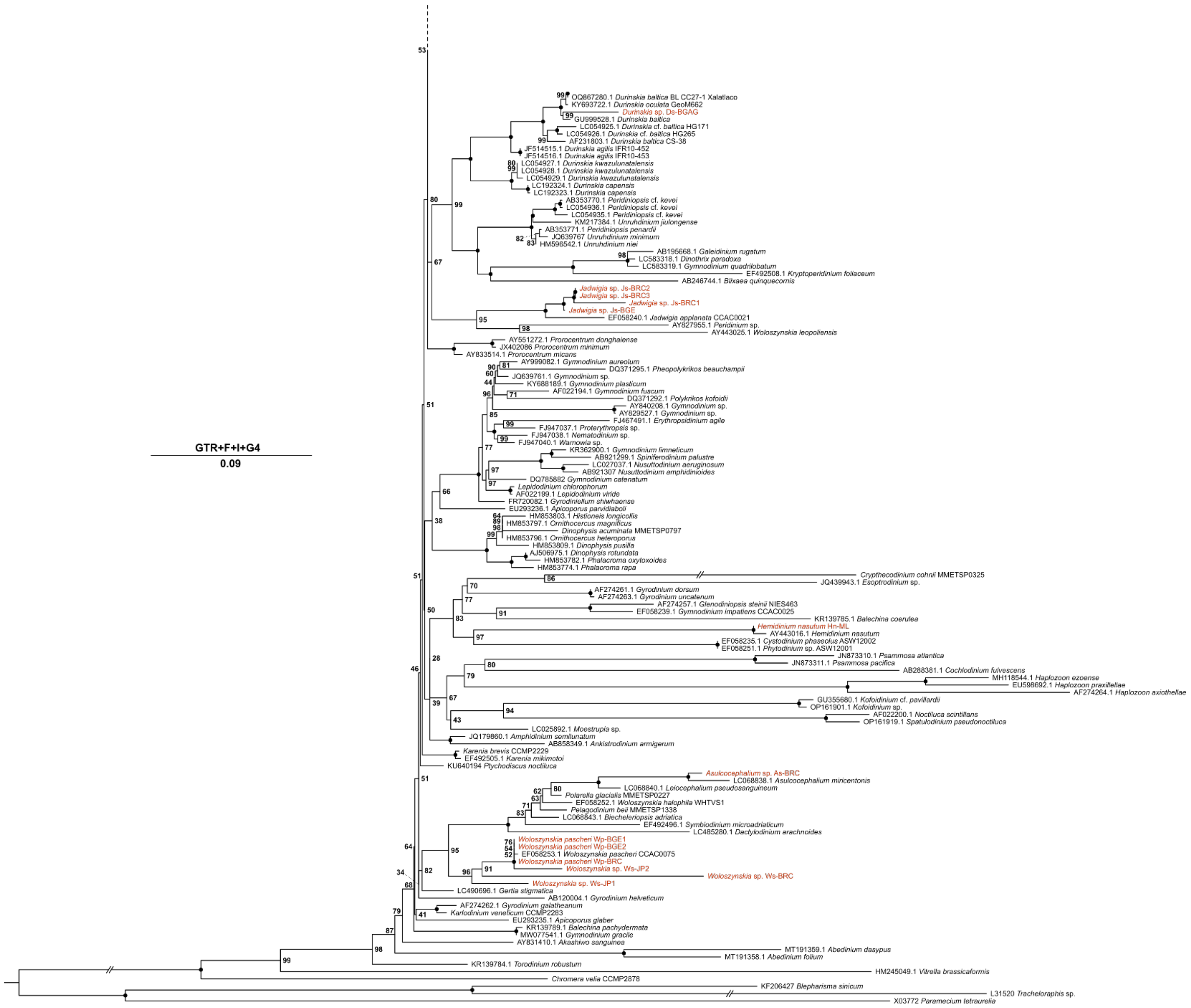

Figure S2 continued.

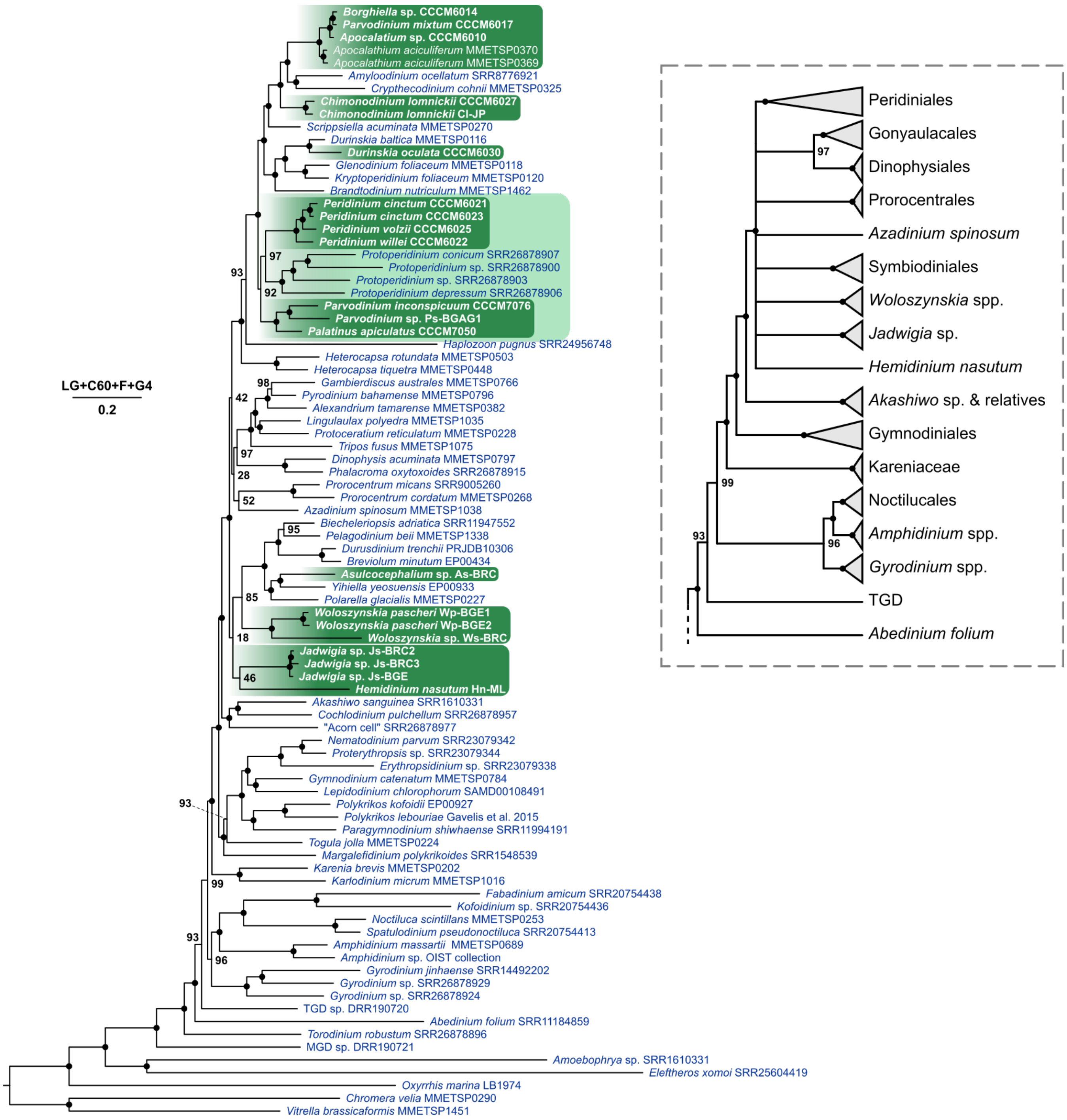

Figure S3. Maximum likelihood phylogeny of dinoflagellates including freshwater lineages. Freshwater clades are highlighted in green and saltwater species are colored blue. Different shades of green highlighting show two different transition scenarios in the *Peridinium*+*Protoperidinium* clade, with light green representing a transition to freshwater by the earliest ancestor of the group followed by a transition back to saltwater by the early ancestor of *Protoperidinium*, while dark green depicts two independent transitions to freshwater by members of this clade. Specific strain or dataset identifiers are shown at the end of each branch label. Inset shows a cladogram interpretation of this tree with node values below 90 collapsed into polytomies. Node numbers represent bootstrap values, with 100 signified by a black dot. The model used to generate the tree is shown above a scale bar showing the estimated number of amino acid substitutions per site. TGD = "Tsuruoka Green Dinoflagellate"; MGD = "Muroran Green Dinoflagellate" [20].

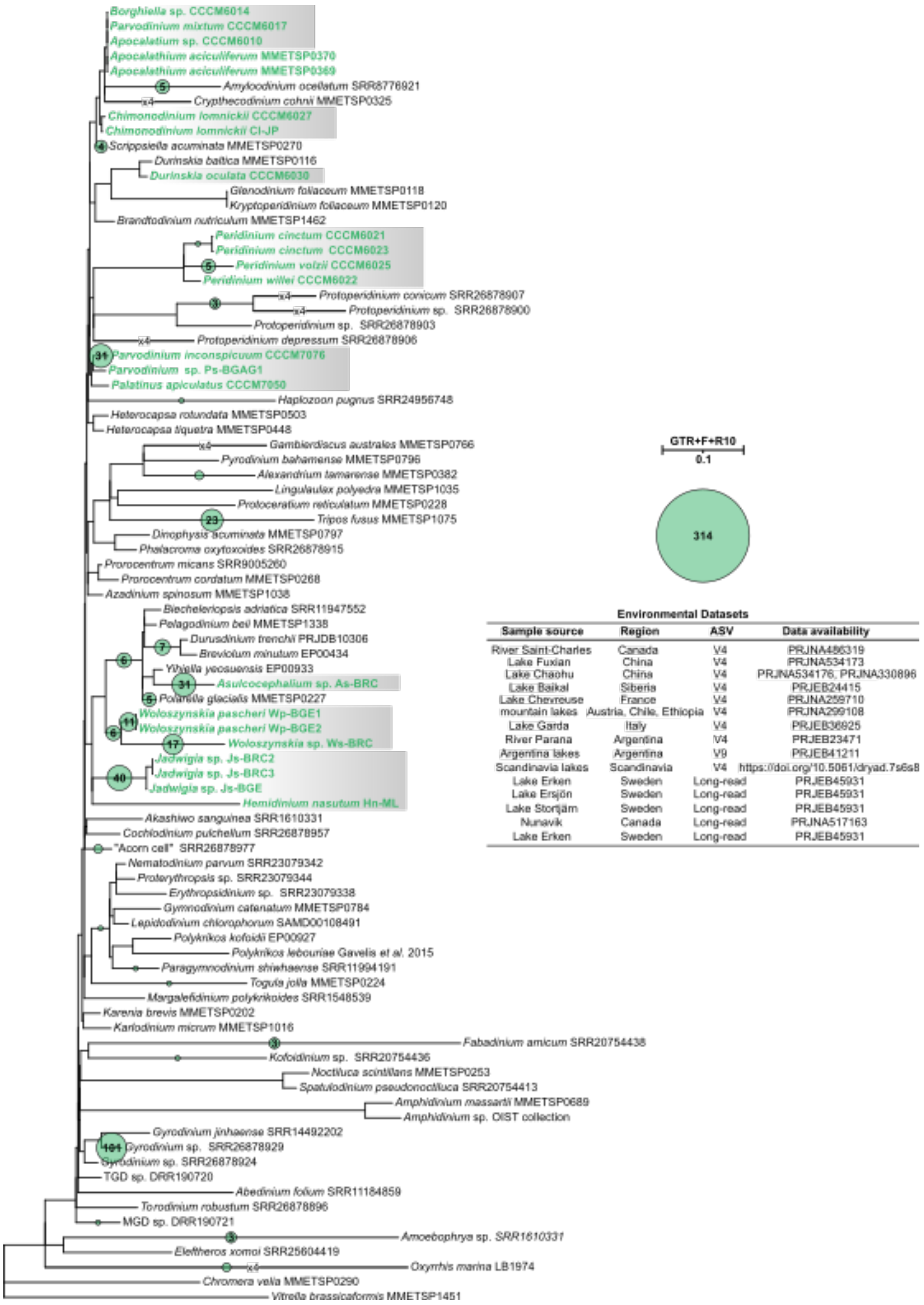

Environmental Datasets

Sample source	Region	ASV	Data availability
River Saint-Charles	Canada	V4	PRJNA486319
Lake Fuxian	China	V4	PRJNA534173
Lake Chaohu	China	V4	PRJNA534176, PRJNA330896
Lake Baikal	Siberia	V4	PRJEB24415
Lake Chevreuse	France	V4	PRJNA259710
mountain lakes	Austria, Chile, Ethiopia	V4	PRJNA299108
Lake Garda	Italy	V4	PRJEB36925
River Parana	Argentina	V4	PRJEB23471
Argentina lakes	Argentina	V9	PRJEB41211
Scandinavia lakes	Scandinavia	V4	https://doi.org/10.5061/dryad.7s6s8
Lake Erken	Sweden	Long-read	PRJEB45931
Lake Ersjön	Sweden	Long-read	PRJEB45931
Lake Stortjärn	Sweden	Long-read	PRJEB45931
Nunavik	Canada	Long-read	PRJNA517163
Lake Erken	Sweden	Long-read	PRJEB45931

Figure S4. Distribution of ASVs from freshwater datasets mapped onto a dinoflagellate 18S rRNA gene phylogeny using EPA-NG. Tree topology was constrained using the tree in **Figure S3** as reference. Freshwater taxa are shown in green; samples from the present study are highlighted with grey boxes. Green circles represent the number of ASVs mapped onto the corresponding branch; numbers are shown for branches with more than one placement. Shortened branches are labeled with the factor by which they were reduced. The model used to generate the tree is shown with a scale bar of estimated nucleotide substitutions per site. The total number of placements was 314. Inset shows the environmental datasets from which ASVs were derived.

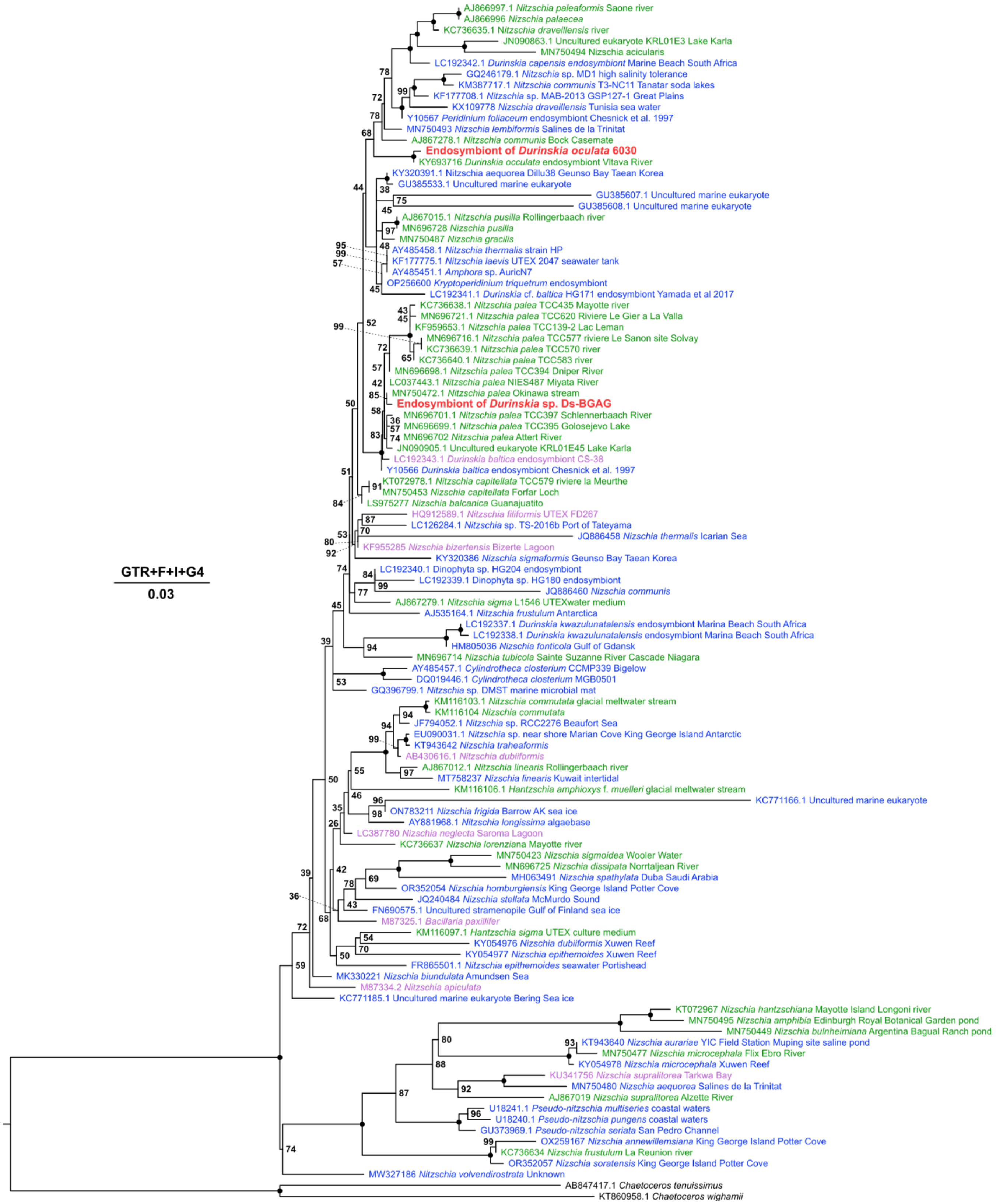

Figure S5. Maximum likelihood 18S rRNA gene phylogeny of *Nitzschia* diatoms including endosymbionts of *Durinskia*. Freshwater, saltwater, and brackish lineages are shown in green, blue, and purple, respectively. Sequences reported in the present study are colored red. Numbers at nodes show bootstrap values with a black dot representing 100. The model used to generate the phylogeny is shown with a scale bar for the estimated number of nucleotide substitutions per site.

References

1. Cho A, Lax G, Keeling PJ. Phylogenomic analyses of ochrophytes (stramenopiles) with an emphasis on neglected lineages. *Mol Phylogenet Evol* 2024; **198**: 1–15.
2. Picelli S, Faridani OR, Björklund ÅK, Winberg G, Sagasser S, Sandberg R. Full-length RNA-seq from single cells using Smart-seq2. *Nat Protoc* 2014; **9**: 171–181.
3. Kolisko M, Boscaro V, Burki F, Lynn DH, Keeling PJ. Single-cell transcriptomics for microbial eukaryotes. *Curr Biol* 2014; **24**: R1081–R1082.
4. Martin M. Cutadapt removes adapter sequences from high-throughput sequencing reads. *EMBnet.journal* . 2011. , **17**: 10–12
5. Bankevich A, Nurk S, Antipov D, Gurevich AA, Dvorkin M, Kulikov AS, et al. SPAdes: A new genome assembly algorithm and its applications to single-cell sequencing. *J Comput Biol* 2012; **19**: 455–477.
6. Haas BJ, Papanicolaou A, Yassour M, Grabherr M, Blood PD, Bowden J, et al. De novo transcript sequence reconstruction from RNA-seq using the Trinity platform for reference generation and analysis. *Nat Protoc* 2013; **8**: 1494–1512.
7. Altschul SF, Gish W, Miller W, Myers EW, Lipman DJ. Basic local alignment search tool. *J Mol Biol* 1990; **215**: 403–410.
8. Poux S, Arighi CN, Magrane M, Bateman A, Wei CH, Lu Z, et al. On expert curation and scalability: UniProtKB/Swiss-Prot as a case study. *Bioinformatics* 2017; **33**: 3454–3460.
9. Burki F, Kaplan M, Tikhonenkov D V., Zlatogursky V, Minh BQ, Radaykina L V., et al. Untangling the early diversification of eukaryotes: a phylogenomic study of the evolutionary origins of Centrohelida, Haptophyta and Cryptista. *Proc R Soc B Biol Sci* 2016; **283**: 20152802.
10. Katoh K, Standley DM. MAFFT multiple sequence alignment software version 7: Improvements in performance and usability. *Mol Biol Evol* 2013; **30**: 772–780.
11. Price MN, Dehal PS, Arkin AP. FastTree 2 - approximately maximum-likelihood trees for large alignments. *PLoS One* 2010; **5**: 1–10.
12. Nguyen LT, Schmidt HA, Von Haeseler A, Minh BQ. IQ-TREE: A fast and effective stochastic algorithm for estimating maximum-likelihood phylogenies. *Mol Biol Evol* 2015; **32**: 268–274.
13. Quang LS, Gascuel O, Lartillot N. Empirical profile mixture models for phylogenetic reconstruction. *Bioinformatics* 2008; **24**: 2317–2323.
14. Hoang DT, Chernomor O, Von Haeseler A, Minh BQ, Vinh LS. UFBoot2: Improving the ultrafast bootstrap approximation. *Mol Biol Evol* 2018; **35**: 518–522.
15. Guiry MD, Guiry GM. Algaebase. *World-wide electronic publication, University of Galway*. https://www.algaebase.org. Accessed 6 Oct 2024.
16. Vaulot D, Sim CWH, Ong D, Teo B, Biwer C, Jamy M, et al. metaPR2: A database of eukaryotic 18S rRNA metabarcodes with an emphasis on protists. *Mol Ecol Resour* 2022; **22**: 3188–3201.
17. Jamy M, Biwer C, Vaulot D, Obiol A, Jing H, Peura S, et al. Global patterns and rates of habitat transitions across the eukaryotic tree of life. *Nat Ecol Evol* 2022; **6**: 1458–1470.
18. Barbera P, Kozlov AM, Czech L, Morel B, Darriba D, Flouri T, et al. EPA-ng: Massively parallel evolutionary placement of genetic sequences. *Syst Biol* 2018; **68**: 365–369.
19. Czech L, Barbera P, Stamatakis A. Genesis and Gappa: Processing, analyzing and visualizing phylogenetic (placement) data. *Bioinformatics* 2020; **36**: 3263–3265.
20. Sarai C, Tanifuji G, Nakayama T, Kamikawa R, Takahashi K, Yazaki E, et al. Dinoflagellates with relic endosymbiont nuclei as models for elucidating organellogenesis. *Proc Natl Acad Sci U S A* 2020; **117**: 5364–5375.
